# Sketches of the Medical Topography of Corfu

**Published:** 1831-01-01

**Authors:** 


### SKETCHES OF THE MEDICAL TOPOGRAPHY OF THE IONIAN ISLANDS.

By the late *John Hennen*, M.D.

IN a former Number, we gave some account of the medical topography of Gibraltar, the key of the Mediterranean Sea, and one of the most impregnable fortresses in Europe, or the world. The Ionian Isles, from their position and importance on the frontier of a resuscitated—we wish we could say a *renovated*, nation of high antiquity and classical renown, are become a colony of great interest to the British nation; and their medical topography deserves a full investigation, since the health of a large number of our troops, as well as of civilians and sailors, is deeply concerned in their salubrity or insalubrity.

### MEDICAL TOPOGRAPHY OF CORFU.

Where SCHERIA's rocks the northern wave divide,

And old CASSOPO greets the straiten'd tide—

Hail, blest PHÆACIA!—*Horæ Ionicæ*.

This island, the present seat of government of the Septinsular Union, has been immortalized by Homer, in the sixth book of the Odyssey, under the names of Scheria and Phæacia—afterwards by that of Corcyra, and now Corfu. It is separated from the coast of Albania by a narrow channel of 21 miles—it is mountainous in character, some of its elevations being 2000 or 3000 feet above the level of the sea—all of them of limestone formation, interspersed with silex. There are only three rivers; but there are several streams that run from marshy villages, round the whole circumference of the island.

Of fresh-water lakes, or rather marshy ponds, there are a great number on this island. They might be easily drained—and, at all events, they are undergoing a natural process of drainage from alluvial deposites. There is one marsh or lake, VAL DI ROPPA, six or eight miles in extent, which bears crops in Summer, and is a sheet of water in Winter. Amongst the casual visitors to this lake for the purpose of shooting, fevers and agues are apt to prevail—and intermittents of the most obstinate kind are found among all the neighbouring inhabitants in the autumnal months. There is a large salt-water lake at Govino, the old Venetian harbour, which is rapidly filling up, and the neighbourhood of which is extremely unhealthy. A large salt-water lake, called Calachiopulo, not more than a mile from the garrison of Corfu.—It is celebrated by Homer as the harbour of the ancient Phæacians, and on its banks were the gardens of Alcinous. It is rapidly filling up. Passing over the canals and salines, we come to the—

### CLIMATE OF CORFU.

This is far from good, according to Dr. Hennen, especially in Summer and Autumn. It is extremely variable at all times, and infinitely inferior to the healthy parts of Italy. Here, as in other places, and even in Italy the thermometer and barometer are poor indices of the atmospherical impressions which the climate produces on the human frame. A person must have felt the winds of the Mediterranean to be able to describe or even imagine them. The northerly winds sweeping over the mountains of Epirus, are cold—while all those from a southerly point, are oppressively hot, accompanied with mist or rain—resembling, in fact, the tramontanes and siroccos of Italy.

"Without affecting the thermometer or barometer in any remarkable degree, the sirocco almost invariably gives the sensation of burning heat and oppresion at the chest, accompanied with languor and a propensity to perspire with the slightest exertion. I have scarcely ever met an individual who was not more or less sensible to these effects; some, who have felt them but slightly on their first arrival, have become exquisitely sensible to them after some time; many can foretel the approach of a sirocco some hours before it begins to blow, by the pecularities of their feelings, and there are few indeed who cannot at once decide, that this wind has commenced, without making any reference to external objects; but it is by the sick and the weakly convalescent that its depressing effects are most severely experienced.

It is a remarkable fact, that wounds and ulcers, and the discharge from mucous surfaces, generally deteriorate during the prevalence of a sirocco; and it is equally certain, that if vaccination, or small-pox inoculations, are performed at this period, they are extremely liable to fail; and if they succeed, the progress of the pustule is often suspended, and it is frequently ten or twelve days in reaching the state usually attained in six or eight.

That the southerly wind in general, and the modifications of it in particular, is unfavourable to the health and spirits of man, is an opinion upon which all classes of persons with whom I have conversed throughout the Mediterranean, are unanimous. All the ancient physicians, who have written upon Mediterranean diseases, from Hippocrates downwards, give their testimony to the same effect, and speak of the pestilential nature of the southerly winds as perfectly familiar. Homer himself, a most accurate observer of nature, when describing the wound inflicted upon Mars by Minerva, a description in which he puts forth all his powers both of imagery and language, represents the god of war as ascending to Olympus in a cloud of

'Vapours blown by Auster's sultry breath,
Pregnant with plagues, and shedding seeds of death.'

ILIAD, *5th Book.*

Whether all the lower animals feel the relaxing effect of the sirocco wind, I know not; horses certainly do, for they sweat sooner, and are more languid than at other times; but on inanimate nature its effects are very obvious, and have repeatedly come under my notice in various parts of the Mediterranean.

The walls of houses, stone floors, and pavements, invariably become moist when the sirocco blows. I have seen the stone floors at Corfu absolutely wet without any rain having fallen; and gentlemen, who have made hygrometrical experiments, state to me, that the instrument has frequently fallen from ten to twenty degrees during the prevalence of this wind.

Although the sirocco is so charged with moisture, vegetables, especially that part of them exposed to it for any length of time, appear quite shrivelled and burnt up, and very frequently they are destroyed altogether. Wine bottled in a sirocco is greatly injured and often destroyed. Meat taints astonishingly soon during its prevalence. No prudent housekeeper ever salts meat at this time; for it either taints at once, not taking the salt, or else it keeps very badly.

Drains and necessaries emit more putrid smells in a sirocco, than at any other period.

No carpenter uses glue in the sirocco, for it does not adhere.

No painter willingly works during its prevalence, for his paint will not dry. I have myself specimens proving this fact, which are now, at the end of three months, nearly in the same state as when painted. The natives assert, that if paint, applied during a sirocco, does happen to dry by intense heat, and a change of wind, it always oozes again on the return of the sirocco: for the correctness of this statement I cannot vouch.

Bakers diminish the quantity of their leaven during the sirocco, as dough is found to ferment sufficiently without it.

It may, perhaps, be difficult completely to account for these peculiarities. The Father of Physic, acute in every thing, is peculiarly so on this subject, and his observations are worthy, not only of attention, but of deep study, because they apply in a peculiar manner to Greece. 'The south wind,' says he, 'blows from places of a like nature with the north: for, coming from the south pole, and breaking through much snow, ice, and hard frosts, or hail, it must needs affect those that live nearest it in the same manner as the north wind; but not the whole country alike that it passes through. For, as it passes through the course of the sun, and under the equator, the moisture is exhaled by the sun, and being dried, becomes rarified, so that it cannot but be hot and dry when it arrives there. In the places, therefore, that are nearest, this hot and dry quality must be imparted, as it is, in fact, in Africa, where the vegetables are dried up, and the inhabitants dried insensibly. For, having neither sea nor river to attract moisture from, it attracts that of animals and vegetables; but when it crosses the sea in this hot and rarified state, it fills the country it falls upon, with much humidity; and therefore, when the situation of a place does not hinder, the south wind must needs be hot and moist.'"

It is fortunate that man can mitigate, in some degree, the effects of the sirocco, by shutting the doors and windows, and confining himself to the house. Dr. H. felt great relief by keeping a large wood fire constantly burning in his room—though to others it appeared unsupportable.

The *soil* of Corfu is generally rich—principally a stiff tenacious clay, very retentive of moisture. Arable land bears a very small proportion to wood-land and pasture. The olive is much cultivated.

*Exhalations*, as might be expected, are abundant here, and of a malarious kind. "There is scarcely a mile in the island free from them, either as they proceed from decaying vegetation, from under-ground moisture, or from the more open swamp." Every shower that falls, if succeeded by heat, is productive of miasmata—but August, and the beginning of September, are the worst months in this respect. The town is more healthy than the country at these periods. The miasmata produce different effects on the natives and on the less seasoned troops—they give rise to intermittents in the former, but severe remittents in the latter.

Passing over vegetable and mineral productions, we may stop a moment on man.

"The upper and front parts of the skull are well developed; the features are, in general, pleasing, and wear an air of intelligence. The complexion, in healthy persons, inclines towards olive; and in some of the females, who are not exposed to the sun, it is clear and white. The complexion of the peasantry is, of course, much affected by the sun. Those who reside in the Lefchimo district in particular, and in the neighbourhood of marshes in general, have a sickly leucophlegmatic cast. The eyes are almost universally brilliant and full, in both sexes, and generally dark-coloured; the teeth good; the hair generally brown or black, and bushy in the men; the beard copious; the figure of the middle standard—sometimes beyond it,—and, if not indicative of strength, it promises activity. The constitution sanguineo-choleric; the gestures vivacious; the gait erect and elastic; and the enunciation voluble and emphatic." 166.

In respect to drains and necessaries, the Corfiots are pretty much on a par with the inhabitants of the Eternal City.

"In lieu of drains, cesspools and necessaries, the Corfiots have substituted either the practice of throwing every species of household offal into the public streets, or depositing them in receptacles called 'Calle Morto.' The calle morto, literally 'stagnant street,' is an enclosure formed at the back of a house, or between the backs of two houses, into which all the filth is discharged; there it often accumulates, until, in process of time, it either rises to the lower windows, and flows into the adjacent buildings, or else bursts the side walls which confine it. Occasionally we see some measures taken to cleanse these Augean depôts, by a train of mules with baskets, which are filled from a breach made in one of the side walls; judging by the appearance and smell of the loading of these animals, the employment of the unfortunate labourer, who works within, must be one of the most disgusting drudgeries to which human nature can be condemned. There are from eighty to ninety of these pestiferous magazines which may be called public, as being common to several buildings, exclusive of those of a minor class, which receive the collected impurities of only single houses." 175.

Even in FLORENCE, which is considered to be one of the cleanest towns to the southward of the Apennines, these reservoirs of filth exist under every house, and are annually or biennially cleared out and sold to the gardeners, to the dreadful annoyance of all northern olfactories that come within the sphere of their effluvia.

The population of Corfu amounts to about seventy thousand souls. The Corfiots are very abstemious, when they fare at their own expense; but when they feast with others, they are "capable of consuming a vast quantity of food, both animal and vegetable, together with copious libations of wine." The estimate for the food of a peasant is about two pounds of Indian corn per day, made into coarse bread, and seasoned by a few leeks, wild herbs, or cloves of garlic, with a little oil and vinegar, and washed down with some water or weak wine, which they denominate *vinetto*. On gala days some caviare, or a morsel of salt-fish adds an additional zest to the meal. On this humble fare, the peasant labours the whole day in the fields. He rises early, swallows a glass of spirits, eats one half of his provisions at noon—the remainder at the close of the day—and he then retires to repose for the night in the same garments which he laboured in during the day! Our labouring classes would soon be in open rebellion on such coarse provender for the belly, and rude couches at night!

In many countries smoaking tobacco may be considered an amusement—in Corfu it is almost an employment. "The pipe is never out of their mouths." The practice, Dr. H. thinks, must necessarily injure the stomach, but he is convinced that it is a preservation against marsh miasmata. The Corfiots have "an irresistible desire for the discussion of news, in the pursuit of which, all their mental and corporeal energies are exerted." Old and young, rich and poor, fly to the town-walls, to the Esplanade, or to some of the adjoining villages, to debate, in groups, on any political event or rumour. It is no matter whether the *bruit* be true or false, if it give him exercise for his tongue. Nor is his body unmoved on the occasion; on the contrary, he distorts it with the most eager and ridiculous gesticulalations. Apoplectic, hysterical, epileptic, and other nervous attacks, are occasioned or renewed by popular meetings, where the greatest excitement obtains.

Morals appear to be at a very low ebb in the Ionian Islands, as well as on most points of the Mediterranean shores. Mr. H. places the national character of the Corfiots as "the very lowest in Europe." Before the English protection existed, "justice was openly sold to the highest bidders —public faith was unknown—and Greek falsehood is proverbial." Their clergy are taken from the scum of the earth, and are illiterate, superstitious, and immoral. One of the principal causes then of degradation and moral decadence in all classes of society is, our author thinks, "the depravity of the clergy." Much, however, must be allowed for "Turkish tyranny and Venetian prostitution" in the demoralizing process.

### Diseases of the Inhabitants.

In Spring, synochus and catarrh; in Summer, bilious remittents; in Autumn, intermittents, rarely typhus; in Winter, pneumonia and other inflammations are the prevailing complaints. Worms are prevalent at all seasons and among all classes. As was mentioned before, the cause which produces intermittents among the inhabitants, gives rise to severe remittents among the troops. A formidable epidemic, supposed to be yellow fever, raged in Corfu about 25 years ago, generally proving fatal on the fifth or sixth day. Bleeding was found to be pernicious—bark, blisters, and tonics were the best remedies. In the ordinary remittents, the native practitioners never use the lancet. After an emetic, the bark is administered.

The state of physic and surgery is at a low ebb in the Ionian Isles. The ordinary routine to qualify for the profession, is an attendance on some old physician or surgeon for a few years, after which a meeting of established practitioners is held, before whom he is examined, and receives a license to practise. There are seven physicians and three surgeons in the town of Corfu. They are stout Brunonians.

As far as we can depend on official returns, not however, perhaps very exact, the prevalence of phthisis in the Ionian Isles is not great. In Corfu, the proportion of phthisis to other complaints among the troops was one in 218 cases. Dr. Hennen is of opinion that the climate of the Ionian Isles only hastens on the fatal termination of confirmed phthisis, but queries whether it may not be advantageous where there is merely a *disposition* to the disease. We wish Dr. Hennen were alive to tell us in what this *disposition* consists? If it consist in the existence of tubercles, small, and not yet taking on the state of mollescence, or as it is more commonly called, suppuration, will a Mediterranean climate retard or arrest the morbid process? We fear it will not. It is curious, however, that of the six islands, three that are notorious for malaria, and, consequently, for intermittents, present fewest cases of pulmonary affection in general. These are Zante, Cephalonia, and Corfu. Ithaca, which is peculiarly dry, has most pulmonary complaints. Santa Maura, although marshy, has had less than Cerigo, which is dry. In respect to pure phthisis, Ithaca presents one in 588—Corfu, one in 212—Santa Maura, one in 211—Cerigo, one in 191—Cephalonia, one in 160—and Zante, one in 147. This table, therefore, does not quadrate with the former observation, as respects phthisis compared with pulmonary complaints in general. Thus Ithaca, which afforded the greatest number of pulmonic inflammations, turned out the smallest number of cases of phthisis. This fact, as far as it goes, militates against the doctrine, that tubercles are the offspring of inflammation.

### TREATMENT OF THE FEVERS OF CORFU.

This relates, of course, to the troops from England. In robust habits, and especially where the men were recently from England, and the inflammatory symptoms ran high, the lancet was used—but with restriction, as it was a precarious remedy in the advanced period of the season, or where the individuals were debilitated. Leeches to the temples, epigastrium, region of the liver, &c. were beneficial. The blood seldom exhibited the buffy coat. Purgatives were found of the utmost importance, as depraved secretions were apt to accumulate to a great extent. "Calomel, in various combinations, had a decided preference." Whenever intermissions, or even remissions were observed, the bark was necessary. "But the sheet anchor, and that which, if it does not supersede, at least most powerfully assists all other remedies, is the early employment of mercury, especially in the more violent cases."

"Almost every individual has his own peculiar methods of administering mercury; in combination with opium; with antimonials, and in combination with cathartic extract; in small doses frequently repeated; in doses of greater extent; in friction, &c. &c. All these may occasionally have their advantages, but however used, where it can be brought fairly to produce its effects on the system, particularly on the hepatic organs, amendment may be looked for in all, and recovery predicted in most cases where the energies of the constitution are not broken down." 232.

Dr. Hennen justly cautions the practitioners in hot climates, and the caution would not be unnecessary beneath our own gloomy skies, to guard against a multiplicity of medicines in fever—bearing in mind, "that the stomach of the patient belongs to an organized and living being, at no time endowed with the passive properties of a chemical vessel, but in a state of disease tremblingly alive to every impression."

### DYSENTERY.

In the severe forms, early bleedings were found to be powerful means of relief, especially leeches to the abdomen, with warm baths and mild purgatives. But, as in fever, mercury was the sheet anchor.

We must now close our notice of the medical topography of Corfu, nor will it be necessary to touch on the other islands belonging to the same group, though their separate consideration will be very useful and interesting to English practitioners in the Ionian Isles, every one of which should possess Dr. Hennen's work. We shall dedicate an article to Malta, the section on which is one of the ablest in the talented author's book.

